# The Neural Correlate Difference Between Positive and Negative Awe

**DOI:** 10.3389/fnhum.2019.00206

**Published:** 2019-06-21

**Authors:** Fang Guan, Sasa Zhao, Shaona Chen, Shi Lu, Jun Chen, Yanhui Xiang

**Affiliations:** ^1^School of Psychology and Center for Study of Applied Psychology, South China Normal University, Guangzhou, China; ^2^UMR 5229, Institut des Sciences Cognitives Marc Jeannerod, CNRS, Université Claude Bernard Lyon 1, Lyon, France; ^3^Department of Psychology, University of Washington, Seattle, WA, USA; ^4^Department of Psychology, Hunan Normal University, Changsha, China

**Keywords:** positive awe, negative awe, voxel-based morphometry, precuneus, insula

## Abstract

Awe is an emotional response to perceptually vast stimuli that transcend current frames of reference. Narrative and experimental work has characterized two distinct variants of awe: positive and negative; however, little is known about the structural neural basis associated with the differences of these two variants of awe. In this study, we investigated the structural neural basis of positive and negative awe underlying individual differences among 62 young healthy adults. Specifically, we assessed the association between regional gray matter volume (rGMV) and the two different variants of awe using the voxel-based morphometry (VBM). A partial correlation analysis was conducted to examine the relationship between rGMV and behavioral positive and negative awe, while controlling for sex, age and total GMV. VBM indicated that positive awe was positively correlated with GMV in the precuneus, and negatively correlated with GMV in the left fusiform and the right calcarine. Negative awe was negatively correlated with GMV in the left and right insula, and the left superior temporal gyrus. These results provide a neural explanation for the differences of these two variants of awe.

## Introduction

Awe as a distinct, profound, and meaningful self-experience, guided by a prototypical emotional approach, that has two core defining features (Keltner and Haidt, 2003). First, awe is induced by stimuli that are vast, including something immense in size, number, scope, complexity, ability, or social bearing (e.g., fame, authority; Keltner and Haidt, 2003; Rudd et al., 2012). Second, awe stimulates a need for accommodation; in other words, when experiencing awe, individuals are forced to adjust their mental schema and alter their understanding of the world (Keltner and Haidt, 2003; Shiota et al., 2007). According to specific appraisal processes, researchers suggested that there are two variants of awe: positive and negative awe (i.e., threat-based or fear-based awe; Smith et al., 2014; Bai et al., 2017; Gordon et al., 2017). Previous studies have explored the concept and influence of awe on individuals’ behaviors (Keltner and Haidt, 2003; Yaden et al., 2016); however, little is known about the neural basis of awe, especially for positive and negative awe. Therefore, we intended to assess the association between regional gray matter volume (rGMV) and two distinct variants of awe using voxel-based morphometry (VBM). Exploring the neural correlate of positive and negative awe may provide us further understanding of the neurological base of the emotion of awe and its subtypes.

Previous studies have revealed that awe involves the complex synthesis of various emotions, including deep feelings of wonder, astonishment, and sometimes tinges of fear (Keltner and Haidt, 2003; Shiota et al., 2007; Yaden et al., 2016). Several researchers considered awe as a self-transcendent positive emotion (Shiota et al., 2006, 2007; Bonner and Friedman, 2011; Rudd et al., 2012; Van Cappellen and Saroglou, 2012; Campos et al., 2013; Smith et al., 2014; Valdesolo and Graham, 2014; Stellar et al., 2015; Bai et al., 2017), while others have argued that further work must be done to investigate negative awe (Bai et al., 2017; Gordon et al., 2017). Theoretical conceptualizations of awe emotion suggested it can be more positive or negative depending on the presence or absence of a threat appraisals process (Gordon et al., 2017). Although positive and negative awe both arise in response to vast and complex stimuli, empirical evidence for the distinctions between positive and negative awe, in terms of its underlying appraisals (Piff et al., 2015; Gordon et al., 2017), subjective experience (Piff et al., 2015; Gordon et al., 2017), physiological correlates (Gordon et al., 2017; Jiang et al., 2018; Guan et al., 2019), were proven. Specifically, positive awe is characterized by greater feelings of calm states, and tonic positive affect (e.g., Oveis et al., 2009; Kok et al., 2013; Krygier et al., 2013), higher appraisals of personal control over the situation. While negative awe experiences are associated with greater feelings of fear and powerlessness, lower in self-control and certainty, and higher in situational control (Piff et al., 2015; Gordon et al., 2017). For example, Piff et al. (2015) explored the downstream effect of negative and non-nature awe experience using an awe-inspiring video, during which participants had similar levels of awe induced from both threatening natural stimuli (e.g., tornadoes, volcanoes) and nonthreatening stimuli, but were imbued with much higher levels of fear and anxiety in negative awe. Therefore, whether to include feeling of perceived threat is key to distinguish positive and negative awe (Gordon et al., 2017).

In addition, experience of awe includes multicomponent processes (Levenson and Ruef, 1992; Keltner and Lerner, 2010; Smith et al., 2014). Emotions involve distinct appraisal processes, subjective experiences, and physiological responses, which give rise to the diverse remarkable outcomes that emotions produce. Keltner and Haidt (2003) considered awe as a primordial emotion; it can be traced to the experiences of individuals from a lower social status and their obedience to authority figures. When associating with authority figures, besides awe, envy may also occur. A VBM analysis indicated that dispositional envy was positively correlated with GMV in several brain regions (e.g., dorsomedial prefrontal cortex, superior temporal gyrus; Xiang et al., 2017). Takahashi et al. (2009) demonstrated that the prefrontal cortex (including the medial prefrontal cortex and the dorsal anterior cingulate cortex) may be extensively involved in the processing of episodic envy. Similarly, we suggested that the cognitive process of awe may also be related to several brain regions (Guan et al., 2018). Previous studies suggested that negative awe experiences were appraised as lower in self-control and certainty than positive awe experiences; and they were characterized by greater feelings of fear (Piff et al., 2015; Gordon et al., 2017; Jiang et al., 2018; Guan et al., 2019). In addition, threat-based awe experiences were associated with physiological indicators of increased sympathetic autonomic arousal (Gordon et al., 2017). Therefore, we speculated that the activation of negative awe may be associated with an automatic threat-detection system that involves brain regions such as the insula (Schienle et al., 2002; Stark et al., 2003). The insula is vitally involved in activating representations of homeostatic states associated with the experience of risk or perceptual awareness of threat (Paulus and Stein, 2006; Xue et al., 2010). Nitschke et al. (2006) found that the insula cortex is crucial for linking emotions to cognitive processes and behavioral responses. As for the activation of positive awe, it may be associated with brain regions during self-reflection. Awe, as a collective emotion, would produce a sense of small self, emphasizing the perception of greatness outside the self, and facilitate the representation of large group identification in the self-concept (Keltner and Haidt, 2003; Shiota et al., 2007; Piff et al., 2015), as well as boosting momentary life satisfaction (Rudd et al., 2012). Functional imaging has linked the precuneus to the processes involved in self-consciousness or self-awareness (Kjaer et al., 2002) and happiness (Sato et al., 2015). A positive relationship has been found between GMV in the right precuneus and the participants’ subjective happiness scores (Sato et al., 2015). A 6-week longitudinal study has shown that mindfulness meditation practice associated detectable precuneus GM increase (Kurth et al., 2014). These results suggested that the precuneus may be positively associated with positive awe. Therefore, we predicted that the precuneus and insula may be the crucial regions for positive and negative awe experiences, respectively. Given the exploratory nature of the current study, we remained open to other possible activation areas in the brain.

The current study aimed to explore the relationships between diverse brain structures and positive and negative awe. A behavioral task was used to induce negative or positive awe experience with awe-inspiring natural scenery with or without fear-based threating stimuli. VBM method was used to identify neural correlates of individual differences in positive and negative awe. We speculated that positive and negative awe would exhibit a structural neural correlation with the precuneus and insula, respectively.

## Materials and Methods

### Participants

Sixty-two young healthy college students from South China Normal University participated in this study [25 men; 37 women; mean age = 20.19 years; standard deviation (SD) = 1.92]. All participants were free of neurological, psychiatric or physical medical abnormalities by self-report according to previous studies (Kong et al., 2015; Xiang et al., 2016, 2017; Guan et al., 2018). This study was approved by the Imaging Center Institutional Review Board of South China Normal University. Written informed consent was obtained from all participants before participating in this research.

### Materials

In previous research, panoramic nature scenery was viewed as an elicitor of positive awe (Keltner and Haidt, 2003; Shiota et al., 2007) and threatening natural disasters could evoke negative awe (Piff et al., 2015; Gordon et al., 2017). In the current study, images associated with positive and negative awe were collected through internet search engine with key words such as “panoramic natural views” and “threatening natural disasters.” Twenty positive awe related natural scenery images such as sunsets and auroras and 20 negative awe related natural disasters images like tornadoes and tsunamis were chosen for further evaluation. Fifteen participants who were not involved in the subsequent behavioral task were recruited to evaluate the images. Twenty positive awe related, 20 negative awe related, and 20 filler related (neutral images, such as river or grass, that were neither associated with positive or negative awe) images were presented in random order. For each image, participants were asked to rate on their arousal (1 = very clam, 7 = very excited), and complexity of the pictures (1 = quite simple, 7 = very complicated) on a 7-point Likert scale. Pictures with rating arousal over five were chosen as target pictures in the behavior task, including 15 positive and 15 negative awe pictures while the filler picture was disregarded. There was no significant difference between 15 positive and negative awe pictures in arousal (*M* = 5.28, *SD* = 0.16; *M* = 5.37, *SD* = 0.09, *t* = −1.99, *p* > 0.05) and the complexity (*M* = 3.70, *SD* = 0.59; *M* = 3.79, *SD* = 0.45, *t* = −0.48, *p* > 0.05).

In addition, to ensure that the above chosen images elicited the desired emotions, we asked the participants rated the extent to which they were feeling: awe, fear and anxiety (Gordon et al., 2017; Guan et al., 2019) using a 7-point Likert-type scale (1 = “not at all,” 7 = “extremely”). The results showed that the negative awe images elicited similar levels of awe as the positive awe images (*M* = 5.77, *SD* = 0.16 vs. *M* = 5.85, *SD* = 0.11, *t* = 1.93, *p* > 0.05) but much higher levels of fear (*M* = 4.65, *SD* = 1.42 vs. *M* = 2.33, *SD* = 0.94) and anxiety (*M* = 4.34, *SD* = 1.38 vs. *M* = 1.91, *SD* = 0.76), which was consistent with previous findings (Piff et al., 2015; Gordon et al., 2017; Guan et al., 2019). All these results suggested that two variants of awe elicited the desired emotions and with balance between arousal and complexity.

### Behavioral Task Procedure

Participants were asked to complete the behavioral task, in which the stimuli were presented using Eprime 2.0 software (Psychological Software Tools, Pittsburgh, PA, USA). The behavior task in this study utilized a within-subject manipulation. Each participant took approximately 25 min to complete the behavioral task, including three blocks with 80 trials each. Each trial began with a fixation point presented at the center of the screen for 500 ms, which was followed by a centrally presented image (positive or negative awe). For each image, each of the eight types of feeling—awe, fear, anxiety, wonder, joy, admiration, feeling of beauty, obedience—would be measured asking to what extent participants experienced those feelings on a 7-point Likert scale (1 = “not at all,” 7 = “extremely”). The image remained visible until the participant pressed one of seven number keys (e.g., 1 = “not at all,” 7 = “extremely awe”). All these feeling types were randomly assigned. The ratings for experiencing awe, fear and anxiety would be recorded for data analysis, and the other five types of feeling measure would serve as filler items to dissemble the purpose of the experiment from participants. In addition, other demographic information such as age and gender were collected.

### MRI Data Acquisition

After the behavior task, an MRI scan was obtained from each participant. The MRI scan was executed by a 3.0-T scanner (Siemens Magnetom Trio, A Tim System) equipped with a 12-channel phased-array head coil at related University, Guangzhou, China. All participants first underwent an MRI scan where they were asked to refrain from head movement, relax, keep their eyes closed and remain awake. The scan comprised anatomical imaging (5 min) and resting state imaging (8 min). The current study only used anatomical imaging data. A three-dimensional magnetization-prepared rapid gradient-echo (3D MP-RAGE) sequence was used to obtain high-resolution T1-weighted anatomical images [repetition time (TR)/echo time (TE) = 1,900 ms/2.52 ms, flip angle = 9°, matrix = 256 × 256, FOV = 230 × 230 mm^2^, Slice thickness = 1.0 mm, Voxel size = 1 × 1 × 1 mm^3^].

### Voxel-Based Morphometry

MR images preprocessing was carried out using SPM8 (Statistical Parametric Mapping, Wellcome Department of Imaging Neuroscience, London, UK) implemented in Matlab 10.0 (MathWorks Inc., Natick, MA, USA). No participant whose images had excessive scanner artifacts or showed gross anatomical abnormalities were excluded. For better registration, the manual method was used to reorient the images to the anterior commissure. T1-weighted anatomical images were segmented into three tissue classes: GM, white matter, and cerebrospinal fluid. We then performed diffeomorphic anatomical registration through Dxponentiated Lie algebra (DARTEL) in SPM8 for registration, normalization, and modulation of the data (Ashburner, 2007). GM images were rigidly aligned and resampled to 1.5 × 1.5 × 1.5 mm^3^ and normalized to a template in MNI152 space. Subsequently, the modulated GM images were smoothed with an 8-mm full width at half-maximum Gaussian kernel. Finally, the modulated images were masked to exclude noisy voxels, using absolute masking with a threshold of 0.2.

### Statistical Analyses

We used SPM8 to perform statistical analyses of GMV. To identify brain regions where regional GMV was associated with individual differences in two variants of awe scores, a linear regression analysis was performed using positive and negative awe scores as the variable of interest. We used age, sex, and total GMV as covariates in the regression model to control for potentially confounding variables. In addition, The AlphaSim program was used to correct for multiple comparisons in AFNI (10,000 iterations) using REST software. The smoothing kernel was calculated with 3dFWHMx. The new re-estimated size of spatial smoothness was larger than original. Using the new smooth size for multiple comparison correction, the voxel-wise intensity threshold was set at *p* < 0.001, and a cluster threshold of *p* < 0.05 (cluster size ≥102) was set. AlphaSim has been widely used in previous VBM studies (Schwartz et al., 2010; Fink et al., 2014; Guo et al., 2014; Kong et al., 2015; Xiang et al., 2016, 2017).

## Results

The results showed that “threatening natural disasters” images associated with negative awe elicited similar levels of awe as “panoramic natural views” images associated with positive awe (*M* = 4.52, *SD* = 1.45 vs. *M* = 4.89, *SD* = 1.27, *t* = −1.50, *p* > 0.05) but much higher levels of fear (*M* = 4.63, *SD* = 1.40 vs. *M* = 2.74, *SD* = 1.25, *t* = 7.95, *p* < 0.001) and anxiety (*M* = 4.41, *SD* = 1.36 vs. *M* = 2.40, *SD* = 1.17, *t* = 8.83, *p* < 0.001).

Supplementary Table S1 reports the mean, SD, skewness, and kurtosis for the positive awe score and negative awe score. All scores for kurtosis and skewness ranged from −1 to 1, indicating normality of the awe data (Marcoulides and Hershberger, 1997), In addition, the negative awe scores were not significantly correlated with participants’ age (*r* = 0.010, *p* = 0.940), sex (*r* = 0.032, *p* = 0.807), or total GMV (*r* = −0.212, *p* = 0.196). The positive awe scores were not significantly correlated with participants’ age (*r* = 0.128, *p* = 0.642), sex (*r* = 0.107, *p* = 0.408), or total GMV (*r* = −0.281, *p* = 0.054).

**Figure 1:**
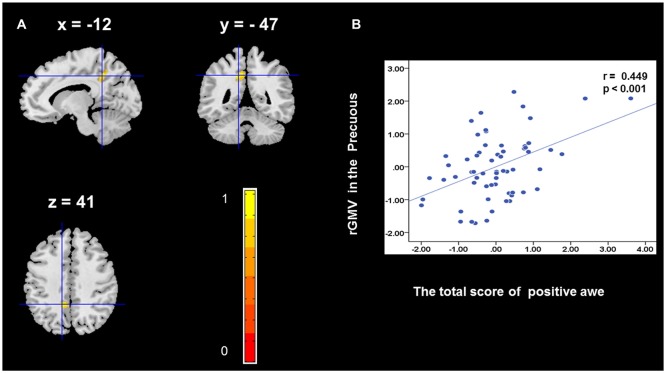
Brain region that is correlated with positive awe. Regional gray matter volumes (rGMV) in Precuneus were positively correlated with positive awe **(A)**. Scatter plots depicting correlations between rGMV in Precuneus and positive awe scores **(B)**.

**Figure 2:**
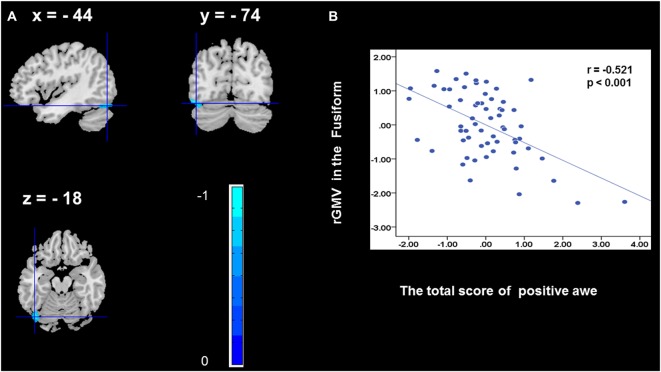
Brain region that is correlated with positive awe. rGMV in Fusiform were negatively correlated with positive awe **(A)**. Scatter plots depicting correlations between rGMV in Fusiform and positive awe scores **(B)**.

**Figure 3:**
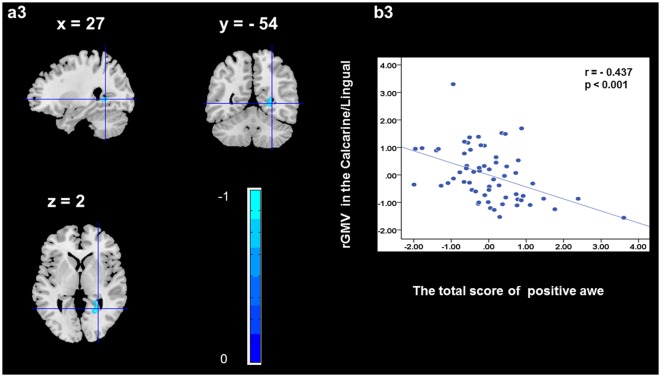
Brain region that is correlated with positive awe. rGMV in Calcarine/Lingual were negatively correlated with positive awe **(a3)**. Scatter plots depicting correlations between rGMV in Calcarine/Lingual and positive awe scores **(b3)**.

**Figure 4:**
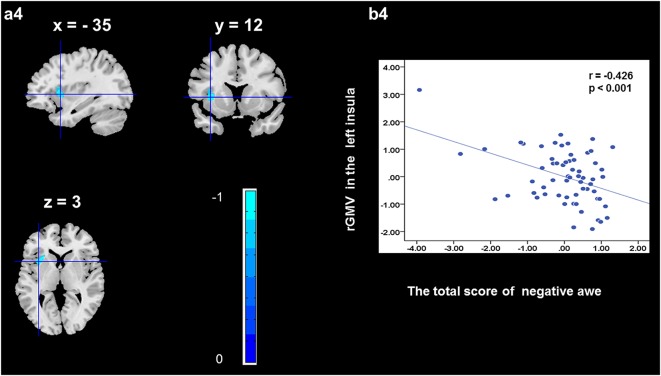
Brain region that is correlated with negative awe. rGMV in Left Insula were negatively correlated with negative awe **(a4)**. Scatter plots depicting correlations between rGMV in Left Insula and negative awe scores **(b4)**.

**Figure 5:**
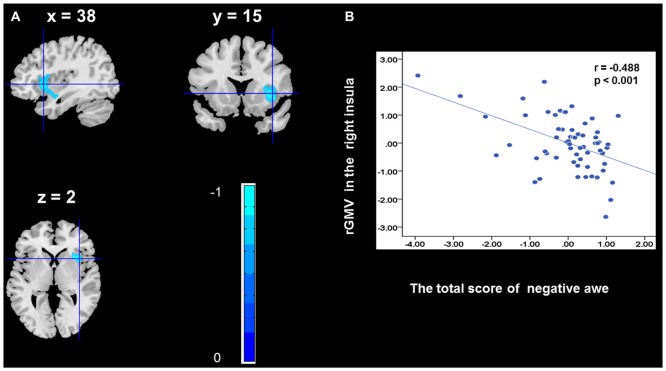
Brain region that is correlated with negative awe. rGMV in Right Insula were negatively correlated with negative awe **(A)**. Scatter plots depicting correlations between rGMV in Right Insula and negative awe scores **(B)**.

**Figure 6:**
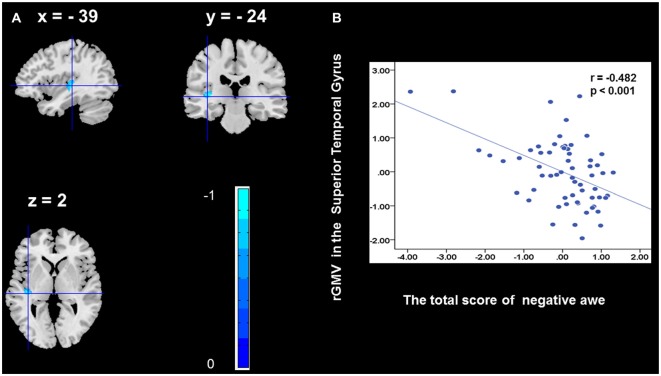
Brain region that is correlated with negative awe. rGMV in Superior Temporal Gyrus were negatively correlated with negative awe **(A)**. Scatter plots depicting correlations between rGMV in Superior Temporal Gyrus and negative awe scores **(B)**.

To determine the neural correlates of awe, we analyzed the correlation between positive and negative awe scores and GMV for each voxel across the brain. After controlling for age, sex, and global GMV, the results of this analysis revealed positive correlation of positive awe with one cluster: the precuneus (MNI coordinate: −12, −47, 41; *r* = 0.449, *t* = 4.10; *p* < 0.001; see Supplementary Table S2, Figure 1), and negative correlations of positive awe with two clusters: the fusiform extend to infer occipital gyrus (MNI coordinate: −44, −74, −18; *r* = −0.521; *t* = −4.38, *p* < 0.001; see Supplementary Table S2, Figure 2), another cluster is the calcarine extend to lingual (MNI coordinate: 27, −54, 2; *r* = −0.437, *t* = −4.06; *p* < 0.001; see Supplementary Table S2, Figure 3). The results of this analysis revealed negative correlation of negative awe with three clusters: the left of insula (MNI coordinate: −35, 12, 3; *r* = −0.426, *t* = −3.77; *p* < 0.001; see Supplementary Table S2, Figure 4), the right of insula (MNI coordinate: 38, 15, 2; *r* = −0.488, *t* = −4.73; *p* < 0.001; see Supplementary Table S2, Figure 5), another cluster is the STG (MNI coordinate: −39, −24, 2; *r* = −0.482, *t* = −4.33; *p* < 0.001; see Supplementary Table S2, **Figure 6**). No other correlations were observed.

## Discussion

This study marked the first attempt, using a VBM analysis, to investigate the neural correlates of individual differences in positive and negative awe experiences. The results of the analyses indicated that positive awe was positively correlated with rGMV in the precuneus gyrus and negatively correlated with rGMV in the fusiform gyrus and calcarine. Negative awe was negatively correlated with rGMV in the insula and STG. This study provides preliminary and novel evidence regarding specific brain structures underlying individual differences in two different variants of awe.

We observed a positive association between rGMV in the precuneus and positive awe. The results suggest that the precuneus plays a critical role in positive awe experiences. According to previous studies, the precuneus is central to the human experience of self-reflections, and aspects of conscious states (Kjaer et al., 2002; Vago and Silbersweig, 2012). Awe, compared with pride and joy, has an impact on the content of the self-concept, that is, it diminishes one’s sense of the individual self (Shiota et al., 2007) but increases one’s sense of the self as part of a greater whole (Piff et al., 2015). Some researchers have considered that awe produces a small self that is central to theoretical characterizations of this emotion (Bai et al., 2017). Moreover, a positive relationship has been found between the volume of GM in the right precuneus and participants’ subjective happiness scores (Sato et al., 2015). This finding is consistent with the increased rGMV within precuneus gyrus of mindfulness practice (Kurth et al., 2014). Previous studies have indicated that experiences of awe could bring people into the present moment, which makes people experience higher life satisfaction (Rudd et al., 2012). In light of these findings, we may reasonably suggest that larger rGMV in the precuneus may be associated with more positive emotions, thus leading to higher levels of happiness or life satisfaction in young healthy adults. Therefore, the result in the current study that rGMV in the precuneus gyrus predicted positive awe significantly may reflect a relationship between positive awe, self-awareness, and subjective life satisfaction.

In addition, we discovered the activation of additional regions, such as fusiform gyrus and calcarine. Previous evidence showed that the fusiform gyrus is critical for perceptual identification of faces (Haxby et al., 2000). We suggested that the precuneus gyrus, fusiform gyrus, and calcarine may have vital functions in the experience of positive awe, which need to be further explored in the near future.

We found that negative awe was correlated with rGMV in the left and right insula. Previous studies have indicated that the insula is involved in interoception (Paulus and Stein, 2006; Jabbi et al., 2008), self-reflection (Paulus and Stein, 2006), decision uncertainties (e.g., risk; Critchley et al., 2001; Preuschoff et al., 2008). The insula plays a vital role in activating representations of homeostatic states associated with the experience of risk and the integration of information relating to bodily states into higher-order cognitive and emotional processes, which in turn influences on subsequent decisions (Xue et al., 2010). In addition, functional imaging experiments have revealed that the insula plays a key role in the experience of a number of basic emotions, including anger, fear, anxiety, disgust, empathy, and sadness (Phan et al., 2002; Schienle et al., 2002; Stark et al., 2003; Critchley et al., 2004; Jabbi et al., 2008; Eckert et al., 2009). This is supported by functional imaging results from Critchley et al. (2004) showing that the structure and function of the right frontal insula are correlated with the ability to empathize with others’ pain. The right anterior insula aids interoceptive awareness of body states, such as the ability to time one’s own heartbeat. Greater right anterior insula GM volume correlates with increased accuracy in this subjective sense of the inner body, and with negative emotional experiences. Moreover, negative awe experienced with stronger feelings of fear and anxiety was associated with the appraisals of threat or risk and sympathetic autonomic arousal (Piff et al., 2015; Gordon et al., 2017). Therefore, these findings may imply that the insula plays a key role in the experience of negative awe and the appraisal of threat-related emotions.

We further observed that negative awe was correlated with rGMV in the STG. The STG plays a vital role in the experience of empathy (Perry et al., 2011; Morelli and Lieberman, 2013), and is associated with perceiving and understanding the emotions or intentions of others. Some fMRI studies (Perry et al., 2011; Morelli and Lieberman, 2013) found that STG is implicated in providing empathy to some positive or negative situations of others. Threat-based awe is guided by the core principle crucial to human experience, that is the ability to detect threat and quickly mobilize the mind and body to respond appropriately to perceived danger (Frijda, 1986; Lazarus, 1991). In a threatening context, individuals could reduce feelings of control and certainty, which ultimately leads to perceptions of powerlessness over their situation (Gordon et al., 2017). Therefore, we suggest that STG may play a critical role in the comprehension of threat-based negative awe experiences of others.

Despite these potential advantages, several limitations deserve consideration. First, we refer to the brain-behavior correlation paradigm of previous researches (Zou et al., 2013; Wang et al., 2014), assessing the association between rGMV and two different variants of awe using the VBM in the current study. Considering the correlational (and not causal) nature of the data, future studies with experimental designs are needed to explore the causal direction of the associations among positive awe, negative awe, and brain structure. Second, the sample was drawn from a college student population which limits the generalizability of our findings, though it is common to choose college students as participants (Takeuchi et al., 2014; Kong et al., 2015; Seger et al., 2015; Xiang et al., 2016, 2017; Guan et al., 2018, 2019). Future studies are necessary to extend our study to include more diverse populations, such as children and adults.

In conclusion, the current study successfully identified potential neural correlates of the two variants of awe using the VBM approach. This study provides preliminary evidence suggesting positive and negative awe experiences are linked to brain regions, such as precuneus and insula, associated with self-representation, emotional regulation, and social cognition from a spontaneous brain activity perspective. In a word, the current study is conducive to further understand the cognitive processing of positive and negative awe.

## Data Availability

Publicly available datasets were analyzed in this study. This data can be found here: https://osf.io/hdmuv/?view_only=4ebfa38db51c486e8f10f19e6da86e9d.

## Ethics Statement

This study was carried out in accordance with the recommendations of Imaging Center Institutional Review Board of South China Normal University with written informed consent from all subjects. All subjects gave written informed consent in accordance with the Declaration of Helsinki. The protocol was approved by the Imaging Center Institutional Review Board of South China Normal University.

## Author Contributions

FG, YX and JC contributed to the experimental design, data analysis, and writing of the initial manuscript. SZ contributed to data analysis. SL and SC coordinated data collection and contributed to translation. All authors approved the final version of the manuscript for submission.

## Conflict of Interest Statement

The authors declare that the research was conducted in the absence of any commercial or financial relationships that could be construed as a potential conflict of interest.
