# Supplementary material for: The Neural Correlate Difference Between Positive and Negative Awe
Source: Front Hum Neurosci. 2019 Jun 21;13:206. doi: 10.3389/fnhum.2019.00206 (PMC6597956; doi:10.3389/fnhum.2019.00206)
Supplement: Supplementary file 1 [file Table_1.DOCX]

Table 1

Descriptive statistics for positive and negative awe (N = 62).

|  | Mean (SD) | Range | Skewness | Kurtosis |
| --- | --- | --- | --- | --- |
| Positive awe | 4.89 (1.27) | 1.29-7.00 | -0.71 | 0.29 |
| Negative awe | 4.52 (1.45) | 1.18-6.96 | -0.45 | -0.60 |

Note: SD: standard deviation
