# Supplementary material for: The Neural Correlate Difference Between Positive and Negative Awe
Source: Front Hum Neurosci. 2019 Jun 21;13:206. doi: 10.3389/fnhum.2019.00206 (PMC6597956; doi:10.3389/fnhum.2019.00206)
Supplement: Supplementary file 2 [file Table_2.DOCX]

Table 2

Significant associations between brain regions and positive and negative awe scores.

| Variables | Brain regions |  |  | MNI coordinates | | |  |  |
| --- | --- | --- | --- | --- | --- | --- | --- | --- |
|  |  | Sides | BAs | x | y | z | Voxel size | Peak-T |
| Positive awe | Positive correlation |  |  |  |  |  |  |  |
|  | Precuneus | L/R | 31 | -12 | -47 | 41 | 208 | 4.10^*^ |
|  | Negative correlation |  |  |  |  |  |  |  |
|  | Fusiform/Infer occipital gyrus | L | 19 | -44 | -74 | -18 | 163 | -4.38^*^ |
|  | Calcarine/Lingual | R |  | 27 | -54 | 2 | 153 | -4.06^*^ |
| Negative awe | Negative correlation |  |  |  |  |  |  |  |
|  | Insula | L |  | -35 | 12 | 3 | 113 | -3.77^*^ |
|  | Superior Temporal Gyrus | L | 22 | -39 | -24 | 2 | 186 | -4.73^*^ |
|  | Insula | R | 13 | 38 | 15 | 2 | 479 | -4.33^*^ |

Note: MNI = Montreal Neurological Institute; L = left; R = right; BAs = Brodmann Areas. ^*^*p*< .001
